# Vertical Lift Systems and Health Outcomes in Community-Dwelling Older Adults: A Rapid Review

**DOI:** 10.3389/phrs.2025.1608955

**Published:** 2026-02-02

**Authors:** Verena Biehl, Denise Abegglen, Adrian Fassbind, Thomas Ballmer, Karin Nordström

**Affiliations:** 1 Institute of Public Health, School of Health Sciences, ZHAW Zurich University of Applied Sciences, Winterthur, Switzerland; 2 Institute of Product Development and Production Technologies, School of Engineering, ZHAW Zurich University of Applied Sciences, Winterthur, Switzerland; 3 Institute of Occupational Therapy, School of Health Sciences, ZHAW Zurich University of Applied Sciences, Winterthur, Switzerland

**Keywords:** aging-in-place, community dwelling older adults, fall prevention, home adaptations, public health, vertical lift systems

## Abstract

**Objectives:**

Despite Switzerland’s aging population increasingly wishes to age in place in familiar homes, many dwellings are not barrier-free. Retrofitting of vertical lift systems as home adaptations in private housing can support aging-in-place but are still rarely installed. Thus, this study examines the association between lift systems in the home environment and health outcomes among community-dwelling older adults.

**Methods:**

A rapid review was conducted to identify and summarize existing evidence. Seventeen studies were included and the results clustered into the categories presence of lifts, usage of lifts and health outcomes.

**Results:**

The review shows that lifts in private housing are scarce and frequently fail to meet the specific needs of users. Usage depends on age, gender, health status, technical and financial aspects. Positive health outcomes include improved mobility, autonomy, safety and quality of life, while lack of lifts can restrict mobility and autonomy.

**Conclusion:**

The results highlight the need for affordable, individualized lift systems supported by advice and policy measures. Such innovations can help reduce inequalities and enable older adults to remain safely in their homes and communities.

## Introduction

In 2022, approximately 1.7 million people aged 65 and older lived in Switzerland, representing 18.7% of the total population. This will almost double by 2050, with 1.1 million people over 80 [1]. While life expectancy rises, aging is accompanied by a growing burden of chronic disease and frailty, increasing the risk of falling [2]. Thus, around one-third over 80 use outpatient care services [3, 4], contributing significantly to the total healthcare expenditures. At the same time, the increasing demand for care is exacerbated by a growing shortage of qualified healthcare professionals [5]. Many health workers experience musculoskeletal pain due to manual patient handling without the use of appropriate assistive devices [6–9]. Combined with generally poor working conditions, these physical strains often drive professionals to leave the field in pursuit of alternative career paths [10].

Thus, demographic aging and the rising demand for care services underscore the need for solutions that enable autonomous “aging-in-place” [11]. This policy refers to the aspiration and possibility of older adults to remain living independently in their familiar home environment or community setting for as long as possible, instead of relocating to institutional care. At its core, the idea assumes that a familiar environment fosters autonomy, identity and social participation, which in turn has positive effects on quality of life in later years [12–15]. In response, international strategies like the WHO Global Strategy and Action Plan on Ageing and Health and the UN Decade of Healthy Ageing (2021–2030) have been developed to promote age-friendly environments. Their focus lies on creating physical, social and policy conditions that enable older people to live safely, barrier-free and independently in their familiar homes for as long as possible [16, 17]. In Switzerland, national organizations such as Pro Senectute, the Swiss Council for Accident Prevention (bfu), or the Barrier-free Architecture Centre (Fachstelle Hindernisfreie Architektur) provide practical standards and recommendations to ensure accessible and adaptable housing for people of all ages and life situations [18, 19].

Yet, many older adults live in inaccessible homes. Two-thirds report their homes are not barrier-free, and renovations are often financially unfeasible [11]. Stairs especially hinder older adults’ autonomous living in their private household [11]. This is particularly relevant considering the large number of privately owned single-family homes in Switzerland. There are approximately one million single-family homes, about 75 percent of which are privately owned [20].

Health promotion and disease prevention, particularly fall prevention, support aging-in-place [21, 22]. One-third of those over 65 fall at least once a year – injuries and fear reduce activity and worsen frailty. Around 60% of those affected suffer injuries [3]. The consequences of falls affect physical, psychological, and social health and have health-economic implications. Measures directed to the individuals are as important as structural measures to shape the health promoting environment [23]. Fall prevention programs are a promising measure to empower older adults and promote physical activity levels, including balance training. The WHO recommends 150–300 min of moderate-intensity activity per week (e.g., brisk walking) or 75–150 min of vigorous-intensity activity, at least two sessions of strength training for all major muscle groups per week, and at least three sessions of balance and coordination exercises per week to help prevent falls [24]. Thus, fall prevention programs often focus on training and advising of older adults to promote their activity level and health literacy. Yet, the structural dimension of fall prevention such as home adaptations (e.g., handrails on both sides of the stairs, bathroom modifications) or technological solutions are often addressed less.

Vertical lift systems offer a neglected yet effective home adaptation. These lifts are primarily designed for the safe and comfortable vertical transport of persons within buildings. In contrast to stairlifts or platform lifts, which are often installed along staircases or as open solutions for short distances, vertical lifts run in dedicated shafts and provide a fully enclosed cabin that ensures safety and convenience for users [25]. If falls prevention is focused mainly on physical activity promotion, it delegates the responsibility to avoid falls mostly to the individuals, neglecting environmental factors [26]. Thus, home adaptations as part of fall prevention strategies need to be better accessible, e.g., information about home adaptations including lifts, comprehensible policy regulations for housing adaptations or easier access to funding options [27, 28].

The demographic change, a shortage of healthcare workers, and inaccessible housing have a significant impact on society. They lead to increased healthcare costs, reduced productivity, greater social isolation and mental health issues, the need for infrastructure changes, and complex policy planning. Thus, this is a call to the public health workforce to find suitable solutions maintaining the wellbeing of older adults enabling autonomous living as long as possible. In order to find promising solutions for aging-in-place, the involvement of several disciplines, sectors, and perspectives is appropriate. Therefore, a project was initiated in collaboration between private industry and the disciplines of engineering, public health and occupational therapy. To the project teams’ knowledge, there is no vertical lift system available on the Swiss market that can be easily and affordably retrofitted into single-family homes while taking both the structural conditions of the building and the individual needs of residents into account. Therefore, the idea was to design a modular, retrofittable vertical lift specifically tailored to the needs of older adults offering a safe and comfortable option to enable aging-in-place for community dwelling older adults. A knowledge gap has been identified regarding the potential impact of vertical lift systems on the health outcomes of community-dwelling older adults. Therefore, a rapid literature review was conducted to inform the development and construction of the first pilot retrofittable vertical lift system.

The following research question is addressed in this study:

What does the literature reveal about the association between lift systems in the home environment and health outcomes of community-dwelling older adults?

## Methods

To identify relevant literature that informs the construction of a modular, retrofittable vertical lift system, a rapid literature review was conducted. The aim was to identify and summarize the existing evidence on the potential health outcomes associated with the use of lift systems in home environments among community-dwelling older adults. The rapid review method is suitable to receive a quick overview of existing literature in the field [29]. Therefore, it is an ideal approach within a running project in collaboration between private industry and engineering to expedite the process for decision-making [30], as was the case in this study. The search was carried out in September 2024.

The PRISMA guidelines informed the rapid review approach and reporting [30]. This rapid review includes the allowances for modifications regarding scope and analysis during the conduct of the rapid review as decisions are made once the nature and volume of the evidence is apparent. If the evidence regarding vertical lift systems and their impact on older adults’ health outcomes is limited, the scope is broadened to include other lift systems and home modifications into the search strategy. Thereby, this rapid review diverges from standard systematic reviews in several respects. First, we limited the search process to two databases, including PubMed and CINAHL, supplemented by manual searches. The manual search combined Google Scholar, screening of reference lists of identified studies and targeted search on websites of professional organizations to capture relevant publications that were not indexed in the selected databases. The quality and the methodological approach of the studies was not considered. We also restricted our review to publications in English and German, which may have led to the omission of relevant studies published in other languages. To streamline the screening process, we dually screened only 25% of abstracts and 50% of full-text articles. Additionally, data collection was expedited by having a single review author conduct data extraction.

In order to systemize the search strategy, the PICO framework was applied: P (population), I (intervention), C (comparison), O (outcome) [31]. We added the S (setting) to specify the search strategy. Various search terms were identified and combined to build the search strategy, which was discussed with the project team including a representative of the private industry and the engineering team. Compound search terms were used to accurately capture the target population of the review, namely, older adults. The I for “intervention” included vertical lift systems, other types of lifts, and home modifications related to lift systems in the home environment. As evidence exclusively focusing on vertical lift systems was very limited, we broadened the scope of the search strategy to encompass also other lift systems. The outcomes were narrowed down to various health-related results such as fall, wellbeing, or quality of life. The search strategy focused on settings in the home environment, like single-family-homes, or multi-family-homes (see Table 1).

**TABLE 1:** Search strategy according to PICOS Schema: Population, Intervention, Comparison, Outcome, Setting. (Rapid review, Switzerland, 2014–2024).

P	Elder*, older people, older adults
I	Stairlift, lift*, elevator, home modification*, assistive technolog*, assisted living, lifting system
C	•
O	Fall*, health, well*being, quality of life, autonom*, independent*, participation*
S	Aging-in-place, home*, household, house, housing

For the study selection, the reference management software Zotero and the tool “Systematic Review Accelerator” were used. Duplicates were sorted out in Zotero. The study selection was guided by inclusion and exclusion criteria. Studies that encompassed a lift (system) as a home adaptation and matched the other search terms were included. The search strategy was limited to publications not older than 10 years to ensure relevance to current technologies of lift systems. Given the rapid review design, this timeframe also allowed for a manageable number of studies while capturing the most pertinent and up-to-date evidence. Studies with no focus on a lift system, or no focus on the home environment as a setting were excluded.

The included studies were transferred to a data extraction table. This table contains information on the author(s) and year of publication, country, population, study design, study objective and relevance of the lift system, and key findings regarding health outcomes related to lift systems. For the content-based data analysis we applied deductive and inductive principles. Categories were retrieved deductively from the research question and categories were inductively added, arising from the studies’ data.

## Results

**FIGURE 1:**
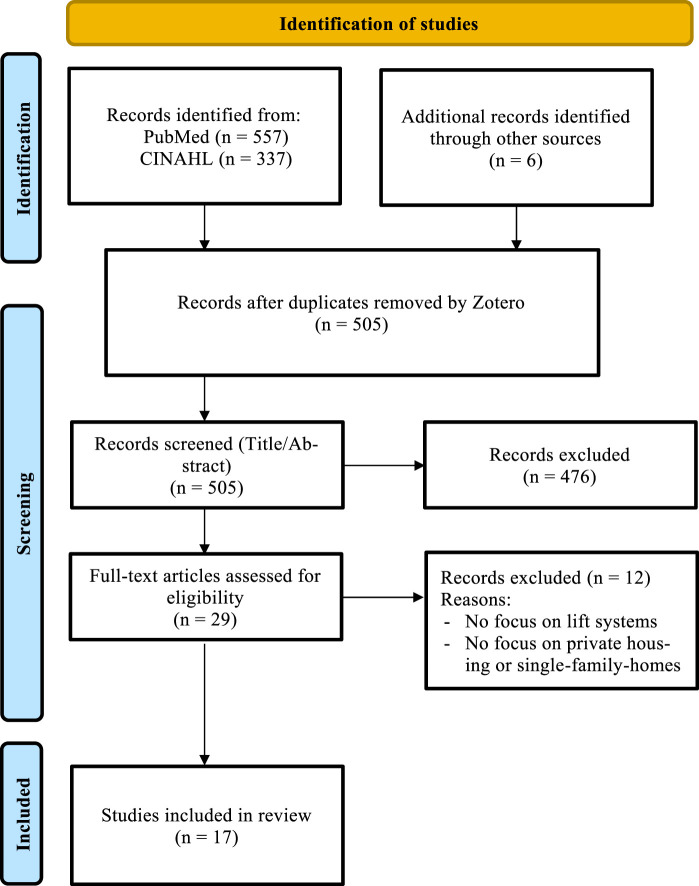
Flowchart of the literature included in the rapid review. (Rapid review, Switzerland, 2014–2024).

The search strategy applied in two data bases revealed 900 articles in total. A first search in the PubMed database yielded a total of 557 hits. An additional 337 studies were identified via the CINAHL Ultimate database. Furthermore, six additional records were identified through a hand search. All 900 references were imported into Zotero, and after removing duplicates, 505 unique records remained. These were screened by title and abstract, resulting in the inclusion of 29 full texts, which were assessed for eligibility. Twelve of these studies were excluded based on predefined criteria (no focus on a lift system, or no focus on the home environment as a setting). In total, 17 studies were included in the final literature review (see Figure 1).

In total, literature on vertical lift systems referred to as home adaptations for older adults in the home environment is very limited. In general, the evidence regarding different types of lift systems and their impact on health of older adults in the home environment is scarce. The identified 17 studies in this rapid review directly or indirectly investigated the relationship between the presence of a lift system as a home adaptation and the wellbeing of older people (see Table 2). The publication period ranged from 2014 to 2024. The countries where the studies were conducted encompass five studies from Sweden [27, 32, 40, 42, 45], three from the UK [35, 37, 38], two from the USA [39, 41], two from Australia [34, 46], two from Spain [43, 44], one each from Germany [33], Turkey [36] and Canada [47]. The study designs were mostly cross-sectional surveys using questionnaires or interviews, as well as longitudinal studies, secondary data analyses, mixed methods studies, qualitative interviews or focus group studies, and a literature review.

**TABLE 2:** Data extraction of included studies in the literature review and results. (Rapid review, Switzerland, 2014–2024).

Author(s) and year	Country	Population	Study design	Study objective and relevance of lift system	Key findings regarding health outcomes related to lift systems
Heller et al. [32]	Sweden	Elderly people	Systematic review	Investigation of the relationship between physical housing features, accessibility of housing and various aspects of health Presence of an elevator	- A higher level of perceived accessibility and usability of the home was significantly associated with higher level of life satisfaction - No association was identified between reduced physical function and older adults living in a multi-story building without an elevator - Having stairs at the entrance increases the risk of going outside independently - Older women living in a high-rise building are less likely to have a decline in I-ADL ((instrumental) activities of daily living) compared to men - No correlation was identified between the decline in I-ADL and living in a multi-story building without an elevator - Stakeholders should focus more on the planning of accessible housing and strive for political solutions at regional, national and international level to support ageing at home
Fotteler et al. [33]	Germany	Elderly people living at home	Cross-sectional study using a written survey	Use and benefits of information, communication and assistive technology for older adults living at home Lift as a potential assistive technology	- A personal lift was used to a greater extent by non-ICT users than ICT users (7.56% vs. 1.61%). This is associated with functional deficits and multimorbidity, which is consistent with the findings that non-ICT users are older and have more comorbidities - Those who had a greater interest in the technology reported that they derived greater benefit from using it
Ainsworth et a [34].	Australia	Elderly people and people with disabilities	Interpretative descriptive interview study	Exploring the experience of home modifications most valued by older adults and people with disabilities Effects of installing a stairlift and other undefined home adaptations	- Participants who appreciated the home changes because they felt they were important to cope in the home generally described positive emotional - Those who were initially ambivalent were surprised by the positive changes - Those who were initially apprehensive often described more disruptive outcomes - The non-involvement of those affected in the home adaptations was seen as negative - Most participants described that the changes enabled them to lead a rich and fulfilling life at home and in the community - After the home adaptation, there were positive outcomes in terms of mental and physical health and wellbeing
Chandola and Rouxel [35]	UK	Older people with significant mobility restrictions	Longitudinal panel study	Investigating whether external and internal housing adaptations reduce the risk of a range of disabilities in older adults Presence of a lift or stairlift	- External housing adaptations (widened doorways, ramps, automatic doors, parking spaces and lifts) help to reduce a range of disability outcomes (falls, poor health and pain) - External housing adaptations reduced the likelihood of not engaging in social activities by 6% and the likelihood of moving by 4%, even for people without mobility impairments - External home adaptations can reduce the risk of falling
Beyazova et al. [36]	Turkey	Elderly people	Cross-sectional multicenter study using an interview survey	Highlighting problems faced by older people in their neighborhoods, buildings and public spaces Presence of a lift and its evaluation	- 76.4% did not have a lift - 10.8% rated the elevator in their building as unsuitable for older people because the (elevator) space is too narrow, the lighting is inadequate, the buttons are not in the right place, or because they have difficulty reading the numbers and signs - (Unsuitable) lifts can cause more falls or cause people to stay at home
Mulliner et al. [37]	UK	Elderly people	Literature review and cross-sectional online survey	To identify the key housing and environmental features associated with the health and wellbeing of older people and to determine preferences for these features through a survey of UK residents aged 55+ The stairlift was considered as part of potential home adaptations	- Home adaptations (such as retrofitting a stairlift) for living in old age were rated as medium to high importance - There is a strong preference for the desire to age at home, which becomes even stronger with increasing age - Age, gender and geographical context had different effects on preferences for housing and environmental characteristics, and education and income were not key factors in housing preferences - The preference for an independent and handicapped-accessible home with an elevator and on one floor increased with age
O’Malley et al. [38]	UK	Patients who have fallen due to a stairlift and are between 40 and 100 years old	Secondary data analysis of a dataset	Investigation of the incidence and pattern of injury in patients diagnosed with a fall from a stairlift Stairlifts	- The overall mortality rate after falling from a stairlift was 15.7% - The most common site of injury (49.2%) was the extremities and the most serious injuries were to the head - The mean fall height was calculated on the basis of the average stair height and amounted to 2.29 m - The fall height is significantly related to the injury of the thorax and the spine
Tural et al. [39]	USA	Elderly people	Sequential mixed-method study using a cross-sectional survey and focus groups	Investigation of changing the environment of stairs with stair mobility aids to support active living for older people Intention for the installation of a stairlift	- The main reasons for using stair mobility aids are product aesthetics, fear of falling and customization of the aid to the environment - Older age and a lower self-reported activity level predict the use of a stairlift - Perceived financial affordability is significantly related to positive attitudes towards stair mobility aids - Possible support from other household members can negatively influence attitudes towards stair mobility products
Andersson et al. [40]	Sweden	People in late adulthood from the age of 55	Cross-sectional study using a questionnaire	The extent to which housing situation preferences are related to age, gender, socio-economic status and geographic area is investigated Lift as a potential home adaptation	- Preferences that increase with age include: 1) the apartment is located in an area where the respondent feels at home, 2) the apartment is handicapped accessible, 3) the apartment has an elevator (if it is higher than the second floor) 4) and the apartment is single-story - Women found it most important to live close to family, have a balcony/terrace, have an elevator and live close to public transportation
Granbom, et al. [41]	USA	Elderly people	Prospective study based on longitudinal data from the representative national health and aging Trends Study	Investigation of whether the accessibility of interior rooms, accessibility of the entrance, safety features in the bathroom, type of apartment and condition of the apartment are related to a move within the community or to a care facility Presence of a lift or stairlift	- The likelihood of moving (to age-appropriate housing) was lower among older adults who lived in a single-story home; had an elevator, stairlift, or had the bathroom, bedroom, and kitchen on the same floor - An apartment with an (stair) elevator can support older adults in aging in place - Installing an elevator can be more challenging and costly than implementing other aids such as handrails
Pettersson et al. [42]	Sweden	Elderly people	Secondary data analysis of two cross-sectional data sets	Assessing the impact of the targeted removal of environmental barriers in the normal living environment Lifts in general	- “Stairs as the only way at entrances” (no elevator/ramp) as an environmental barriers to be eliminated were significantly less common in apartment buildings and single-family homes - The results show that of the current 2.3 million apartment buildings in Sweden, more than 1 million have entrances with steps without a ramp or elevator. The proportion and actual number of single-family homes is even higher
Pérez-Hernández et al. [43]	Spain	Elderly people living at home	Prospective cohort study using telephone interviews and home visits	Investigation of the influence of poor living conditions on the functional status of older adults Presence of a lift	- There is an increased risk of reduced physical activity due to the lack of a lift
Slaug et al. [27]	Sweden and Germany	Elderly people living alone and at home	Simulation of policy changes regarding the elimination of hindering housing features in Sweden and Germany by means of a survey with a 12-month follow-up	Testing the hypothesis whether the new directive to remove potentially disabling housing features can maintain older people’s independence in relation to I-ADL and thus reduce the overall need for home services Lift as a potential environmental barrier	- The simulations predicted that new measures to remove potentially disabling housing features could improve I-ADL performance of older people and reduce the need for home-based services - The results suggest that policy change can contribute to positive effects in terms of I-ADL dependency among older people and a reduction in societal costs
García-Esquinas et al [44]	Spain	Elderly people living at home	Cross-sectional study using telephone interviews and physical examination	Relationship between housing conditions and physical functional limitations in older adults Presence of a lift	- The lack of an elevator in apartment buildings can prevent some older adults from leaving their homes, which is associated with higher mortality in patients with heart failure - Not leaving the home may be associated with an increased risk of cardiovascular disease or diabetes when physically inactive. This may also limit access to a balanced diet. - Not leaving the home can increase the risk of feelings of loneliness and depression
Granbom et al. [45]	Sweden	Elderly people	Secondary data analysis of three datasets	Identify the environmental barriers that cause the most accessibility problems for subgroups of the ageing population with different combinations of functional limitations Presence of a lift	- Stairs are very often the only way (no elevator/ramp) at entrances, which confirms the social relevance of supporting active and healthy ageing - The recent government commission on housing for the ageing population in Sweden has proposed that owners of apartment buildings should receive financial support of up to 50% of the cost of installing elevators in existing apartment buildings
Aplin et al. [46]	Australia	Clients and their families who have made changes to their living space	Qualitative descriptive study	The aim of the study was to investigate the effects of changes to housing on the experiences of clients and their families in their homes Lift as a potential home adaptation	- The changes (including the installation of the lift) made everyday life easier and the participants were able to live in their home without strain. They were able to carry out their daily activities more easily - The physical dimension of the home, in particular the living environment, was more often negatively than positively influenced by the adaptations at home - Public accessibility standards (e.g., building regulations) and the limitations of service providers or restricted budgets - Standard procedures and guidelines were constraints, so some participants were not able to bring their home to a level they considered acceptable - Participants felt the work was unfinished, with missing towel rails, unpainted areas and elevators and ramps that needed to be covered with awnings
Mattie et al. [47]	Canada	Users of the lift: occupational therapists and wheelchair users	Description of technical development and user experience inclusion in the design process	Development of an integrated stairlift “ARISE” for the house entrance Newly developed stairlift	Feedback from occupational therapists - In terms of cost aspects, the price must be manageable and financing options must be available - Safety: the lift must not be dependent on an external power supply, even if it is self-powered - The physical and mental health of the end users and the voice of the care staff are important factors when deciding on home access options - The design must be ‘inclusive’ so that there is no stigma associated with accessible products Feedback from end users (wheelchair users) - Maintenance costs must also be low - There must be enough space to turn around and possibly be accompanied by a caregiver - In terms of aesthetics, the lift should look “unobtrusive” and “blend in” with the overall appearance of the building

### Results on Lift Systems and Health Outcomes for Community Dwelling Older Adults

The results were clustered in different categories that support answering the research question. Due to the limited evidence identified, the derived inductive categories include “presence of lifts,” and “usage of lifts,” and the deductive category was “health outcomes of lifts.”

#### Presence of Lifts

The limited literature referring to vertical lift systems in home environments broadened the scope of the data analysis and thereby helped deriving information about the situation of lift systems in general. The identified studies were not exclusively referring to single-family homes but also included multi-family homes. In total, the presence of a vertical lift in home environments are rather scarce. In Turkey approximately 25% (Turkey) of households were reported to use lift systems [36]. In Sweden about half of all multi-family homes have stairs at the entrance without a lift [42]. Beyond this scarcity of access to vertical lifts in home environments, existing lifts are often unsuitable for people with disabilities, e.g., too narrow, poor lighting, or small lettering [36].

#### Usage of a Lift

The use of a (stair) lift is dependent on different socioeconomic determinants. Persons of older age are more likely to use the lift [37, 39, 40]. Also, gender influences the likeliness to use a lift with women being more willing to use lifts [40]. Moreover, financial affordability [39, 41, 47], low maintenance costs [47], and the effort and expenses of retrofitting the home environment influences the usage of a lift [41]. Beyond the socioeconomic determinants, the persons’ health and activity status influence the likeliness of using a lift. This is Information and Communication Technology (ICT) affinity [33], a lower self-reported activity level and fear of falling [39]. Moreover, some structural-technical aspects are relevant to potential lift users: The aesthetic appearance [34, 46, 47], sufficient space, safety and low noise [47]. Ideal is the inclusion of users in the planning process [34] and individual adaptation of the lift to the users’ needs [39].

In addition to these individual factors, further social structural factors are relevant for older adults’ lift use. On the one hand, the attitudes of relatives [39] are highly relevant. On the other hand, some studies highlight the adjustment of guidelines for home adaptations as a central public health measure to facilitate aging-in-place and, thereby, reducing societal costs in the long-term [27, 45].

#### Health Outcomes of Lifts

Diverse positive and negative outcomes on health and wellbeing of community dwelling older adults related to the presence of lift systems were described in the included studies.

Positive health outcomes are related to better usability of living spaces and higher mobility due to lift systems [43]. This in turn leads to a sense of autonomy [46] and enabling older adults to stay in their homes [41]. Moreover, lifts are associated with a perception of improved safety and a reduction of the fear of falling [46], reduced disability outcome following potential falls [35], and an increased physical and mental health and wellbeing [34]. Overall, better quality of life [32] and a rich and fulfilling life at home and in the community [34] is associated with the presence of a lift system in the home environment of community dwelling older adults.

Negative health outcomes were described related to stairlifts, namely, an increased risk of falling mainly accompanied by fractures of the extremities (49%), in rare cases with injuries to the head and spine, or even fatal outcomes [38].

Further negative health outcomes described in the studies were related to the accessibility of the home environment only via stairs. The absence of a lift is associated with lower mobility and lower engagement in social activities [32, 35, 45]. This in turn may lead to loneliness, depression, a higher risk of cardiovascular disease and restricted balanced diet [44].

## Discussion

The literature review aimed to identify and summarize existing evidence on the potential health outcomes of lift systems among community-dwelling older adults to inform an ongoing interdisciplinary project on the development of a retrofittable vertical lift system. The findings reveal a clear research gap concerning the health-related outcomes of vertical lift systems, specifically in single-family-homes. While multiple studies identified positive outcomes, such as increased quality of life, increased autonomy, increased mobility, improved physical and mental health and greater wellbeing, few focused specifically on vertical lifts in private homes. Although these benefits suggest significant potential for supporting aging-in-place, successful implementation depends on several factors. Socioeconomic variables such as age, gender, and income, along with individual preferences regarding design and aesthetics, influence adoption. Technical complexity, renovation costs, perceived safety, and the attitudes of family members also shape decisions. Aversive attitudes among cohabitants or relatives may hinder uptake, underscoring the need for inclusive planning processes. Beyond these aspects, the importance of policy alignments for home adaptations are a crucial public health measure to facilitate justification, funding and acceptance of home adaptations in community dwelling older adults living in single-family-homes.

The increasing demand for age-appropriate housing driven by demographic aging, highlights the urgent need for innovative, person-centered solutions [11, 48]. In this context, the limited research on the implementation of vertical lift systems in single-family homes represents a significant public health gap. Most studies identified in this review examined general home adaptations but only marginally addressed vertical lift systems, resulting in a limited number of relevant search results. This suggests that retrofittable vertical lifts remain underexplored in scholarly discourse as a viable aging-in-place strategy. Additionally, many studies did not clearly differentiate between types of lift systems such as stairlifts versus vertical lifts, which complicated the interpretation and applicability of the studies’ results [27, 32, 33, 35, 36, 40–43, 45, 46]. One study even reported fall incidents related to stairlift use [38], underscoring potential safety concerns. In contrast, vertical lift systems may offer improved safety and accessibility for users. Another study emphasized the development of a specialized stairlift, incorporating input from occupational therapists and wheelchair users [47], highlighting the value of user involvement in design processes. These findings point to the importance of integrating users and professional stakeholders into the planning and implementation of lift systems. Interdisciplinary, human centered design are particularly promising for successful adoption in single-family-homes [49, 50]. Further research, especially longitudinal and comparative studies, is needed to evaluate health outcomes and distinguish the benefits of vertical lifts from stairlifts in promoting autonomous living among older adults.

The findings of this literature review also revealed important knowledge gaps concerning the influence of relatives and healthcare professionals on the decision to install or retrofit lift systems. Some studies suggest that negative attitudes among cohabitants or family members can discourage older adults from pursuing lift installations, even when such adaptations would support their autonomy and safety [39]. More broadly, literature on home adaptations indicates that both family members and health professionals are key stakeholders in the decision-making process [28, 51]. Their support, or lack thereof, can significantly shape the willingness of older adults to invest in modifications. This underscores the need for further research into the perspectives, knowledge, and attitudes of these influential actors. Specifically, it is essential to explore their understanding of available home adaptation options, awareness of relevant regulations and financial implications, and level of trust in construction providers. Gaining deeper insight into these factors would help develop targeted, evidence-based information strategies tailored to different stakeholder groups. Improved communication and guidance for families and professionals could reduce uncertainty, enhance trust, and ultimately facilitate more timely and appropriate adaptation decisions. Such efforts would provide a crucial foundation for promoting lift systems and other age-friendly modifications as part of a broader public health strategy to support safe, independent living for older adults.

Given that over half of older adults reside in homes accessible only via stairs—particularly in private residences and smaller apartment buildings [2, 15], the absence of lifts represents a major barrier to mobility and independence. As this review and related literature demonstrate, such restrictions are closely linked to a range of negative health outcomes, including increased risk of falls, social isolation, and reduced autonomy [11, 28, 51, 52]. To address these challenges, public health strategies must prioritize measures that promote the retrofitting of vertical lift systems in existing housing stock. Retrofitting efforts should be guided by principles of accessibility, affordability, and user-centered design. Adaptations must be unobtrusive and harmonize with the home environment to avoid stigmatization or the impression of medicalization. Preserving the familiar and personal character of the living space is essential for maintaining quality of life and psychological wellbeing among older adults [28, 34, 46, 47, 51]. In this context, aesthetic integration and design sensitivity are not secondary concerns but central elements of successful implementation. Developing lift solutions that are both functional and discreet can improve user acceptance and long-term satisfaction. Moreover, interdisciplinary collaboration between architects, engineers, health professionals, and users themselves is crucial to designing systems that meet diverse needs and preferences.

To maintain quality of life for an aging population and effectively support aging-in-place strategies, stronger political commitment to retrofitting private housing, particularly single-family homes, is essential. Revising and aligning home adaptation guidelines within the political agenda represents a crucial step toward improving health equity among older adults. Financial barriers remain a primary obstacle to the widespread adoption of lift systems. This review identified key cost-related factors influencing uptake, including overall affordability [39, 41, 47], low maintenance costs [47], and the perceived financial and logistical burden of retrofitting [41]. These findings are consistent with broader literature, which emphasizes that financial concerns are central to older adults' housing decisions and willingness to invest in home modifications [28, 51]. In addition to economic constraints, older adults often cite administrative complexity such as unclear building regulations, fragmented funding opportunities, and bureaucratic hurdles as significant deterrents to home adaptation [28]. These factors can result in delays or complete avoidance of essential modifications, despite the clear health benefits associated with improved accessibility. Consequently, revising home adaptation policies and guidelines to include transparent, accessible, and equitable funding mechanisms including funding options should be a top priority within public health, health promotion and disease prevention strategies, particularly in the context of fall prevention and aging-in-place. Such policy adjustments would not only reduce structural barriers but also promote broader awareness and acceptance of home adaptations, including vertical lift systems, as standard elements of age-friendly living environments.

Unlike multi-unit residential buildings, private homes in Switzerland are not subject to mandatory accessibility standards such as barrier-free construction (SIA 500) or the Disability Discrimination Act (BehiG) [53]. In the case of detached, single-family homes, only a notification obligation applies, with no formal requirement for building permission. While this legal flexibility allows older adults a greater degree of autonomy when modifying their homes, it also presents challenges in terms of standardization, oversight, and access to financial support. Crucially, funding opportunities for structural adaptations in private homes remain limited and inconsistent. Invalidity Insurance (IV) and supplementary benefits (EL) typically provide support only in cases of medically certified disability, leaving a substantial portion of older adults—particularly those with age-related functional decline but no formal diagnosis—without access to financial aid. General health insurance does not cover home modifications, further limiting options for preventative adaptations. Non-profit organizations such as Pro Senectute or the Swiss Council for Accident Prevention (bfu) can offer guidance and, in some cases, partial financial support However, these services vary widely across cantons, leading to fragmented and unequal access to home adaptation funding throughout the country [11, 28]. This regional disparity underscores the need for policy alignment across federal, cantonal, and municipal levels to ensure equal access to home adaptation support nationwide. Stronger national policies could establish clear standards for aging-friendly housing and mandate the retrofitting of vertical lift systems in existing residential structures where feasible. Such regulatory measures would not only promote greater equity but also advance broader public health goals by supporting aging-in-place, preventing falls, and reducing the long-term burden on health and social care systems [28, 39, 45].

### Pilot Construction of a Retrofittable, Individualized Vertical Lift System

The findings of this literature review inform the design of a retrofittable, individualized, and cost-effective vertical lift system, developed in collaboration with private industry, engineering, public health and occupational therapy. The concept is outlined below.

**FIGURE 2:**
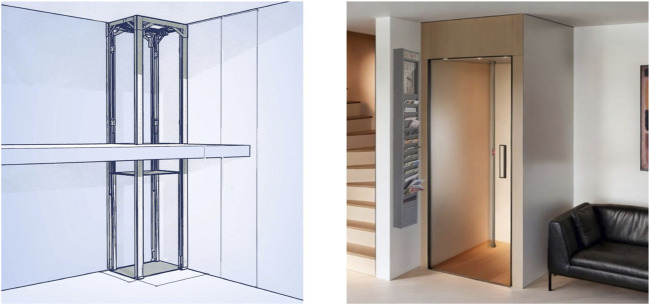
Construction of the vertical lift system as a model (left) and a visualization in the home environment (right). (Rapid review, Switzerland, 2014–2024).

The vertical lift system offers several advantages over conventional stairlifts, which are associated with various disadvantages for users, such as the risk of falling when getting on and off the lift and tipping over onto other walking aids [38], reduced stair width, limited installation of handrails or changes to the living atmosphere, including stigmatization of the disability. The modular system of the vertical lift system considers the needs of the user and their living environment and can be individually installed in the living or sleeping area within a very short time if necessary. After a brief static test, the ceiling opening of less than one square meter is cut out, considering wooden floors or concrete ceilings and underfloor heating. The basis is the upper central suspension with a helical-bevel brake motor with double brake and counterweight balancing system. The flat belts for raising and lowering the cabin are characterized by maximum strength by combining high-strength steel cables with high-tech polymers and thus represent a maintenance-free, low-noise and cost-effective solution. Further technical requirements can be adapted to individual needs, such as controls via voice command or facial recognition, as well as emergency call systems. The lift system is depicted in Figure 2 [54].

### Limitations

This rapid review has several limitations. The use of a simplified search strategy with only two databases and limited time, which is typical for rapid reviews, may have limited completeness and depth. This increases the risk of missing relevant studies as well as the risk of bias and errors. Furthermore, some of the included studies did not focus exclusively on single-family homes but also referred to multi-family buildings or general housing contexts. This limits the direct transferability of the findings to the specific target setting of private homes. The review was further limited to studies published in English and German, which may have excluded relevant evidence in other languages.

Despite these limitations, this review also demonstrates important strengths. It offers practical insights into a topic of high relevance for promoting aging-in-place of community dwelling older adults. The collaboration between private industry, engineering, public health and occupational therapy contributes valuable interdisciplinary perspectives. Moreover, research on modular vertical lift systems for private homes is still rare because such solutions have not yet become widely accessible and affordable for most households.

### Conclusion

The current literature suggests a promising yet underexplored association between the use of vertical lift systems in private homes and the health outcomes of community-dwelling older adults. While initial findings point to potential benefits in promoting autonomy, mobility, and fall prevention, robust empirical evidence, especially longitudinal and comparative studies are still lacking. This gap highlights a critical need for further research that systematically investigates health impacts of vertical lift systems, particularly in comparison to other assistive technologies such as stairlifts.

Key conclusions can be drawn for the planning and implementation of such systems. Integrating older adults as end-users, their relatives and family, alongside health professionals, architects, and construction stakeholders, into the design and decision-making process is vital. Interdisciplinary, human-centered design approaches have shown potential in enhancing the usability, acceptance, and effectiveness of assistive home technologies, especially in single-family-homes.

Furthermore, the attitudes and knowledge of caregivers, relatives, and professionals regarding home adaptations, including the complexities of regulations, costs, and trust in construction companies, emerge as important factors influencing implementation. Targeted information strategies and clearer guidance tailored to these groups are necessary to foster informed decision-making and wider adoption of vertical lift systems as effective home adaptations.

Crucially, legislative and financial barriers remain significant obstacles in retrofitting home environments. Inconsistent regulations and fragmented funding schemes across Swiss cantons limit equitable access to home adaptations. Addressing these systemic issues, such as harmonizing retrofitting laws and promoting financial support through public policy, should be prioritized as a public health measure both internationally and in Switzerland. Integrating home adaptation policies into health promotion, fall prevention, and aging-in-place programs can significantly contribute to health equity and quality of life for older adults. This is a call to stakeholders in the field of older adults, scholars and (health) professions to address the structural factors of health promotion and especially of fall prevention.

In sum, advancing the integration of vertical lift systems in private homes requires a multifaceted approach, anchored in evidence, user-centered planning, and supportive regulatory frameworks, to effectively support aging-in-place and promote health equity for older adults. The pilot construction of a modular, retrofittable and individualized vertical lift system as part of this project is a promising innovation to foster aging-in-place and therefore of major public health relevance.
